# Prevalence of Hypogonadism and Associated Risk Factors among Newly Diagnosed ART Naïve HIV-Infected Males in Mwanza, Tanzania

**DOI:** 10.1155/2024/9679935

**Published:** 2024-03-05

**Authors:** Shabani Iddi, Haruna Dika, Benson Kidenya, Samuel Kalluvya

**Affiliations:** ^1^Department of Physiology, Weill Bugando School of Medicine, Catholic University of Health and Allied Sciences, Mwanza, Tanzania; ^2^Department of Biochemistry, Weill Bugando School of Medicine, Catholic University of Health and Allied Sciences, Mwanza, Tanzania; ^3^Department of Internal Medicine, Weill Bugando School of Medicine, Catholic University of Health and Allied Sciences, Mwanza, Tanzania

## Abstract

**Background:**

Hypogonadism is frequent among HIV-infected males and might have significant clinical impact leading to sexual impairment and metabolic derangement. There is limited information about the magnitude of hypogonadism and its associated factors among people living with HIV in Tanzania. We aimed to determine the prevalence of hypogonadism and associated risk factors among newly diagnosed ART naïve HIV-infected men in Mwanza, Tanzania.

**Methods:**

Newly diagnosed ART naïve HIV-infected men were enrolled at Voluntary Counseling and Testing Centres of four selected hospitals in the Mwanza region and subjected to thorough clinical and general physical examination including anthropometric measurements. A prestructured questionnaire was used to collect sociodemographic characteristics and clinical data. Serum total testosterone, follicle-stimulating hormone, luteinizing hormone, and estradiol were estimated. Serum total testosterone <300 ng/dl or testosterone >300 ng/dl with high LH and FSH (compensatory hypogonadism) was taken as markers of hypogonadism. Data were analyzed using STATA version 15.

**Results:**

Of the 388 enrolled participants, hypogonadism was found in 47.9%, with secondary hypogonadism (83.9%, 156/186) being the most frequent form. Logistic regression analysis showed a significant association between hypogonadism and CD4+ count (OR 2.0; 95% CI 1.1–3.6; *p*=0.022), decreased libido (OR 1.6; 95% CI 1.1–2.4; *p*=0.024), age of above 46 years (OR 2.3; 95% CI 1.1–4.6; *p*=0.023), herbal medicine use (OR 2.4; 95% CI 1.5–3.9; *p* < 0.001), WHO clinical stage 3 (OR 2.7; 95% CI 1.4–5.2; *p*=0.003), and weight loss (OR 1.8; 95% CI 1.1–3.0; *p*=0.016).

**Conclusion:**

Hypogonadism was found in nearly half (47.9%) of ART naïve HIV-infected men. The majority (83.9%) had secondary hypogonadism. There was a significant association of hypogonadism with older age, herbal medicine use, weight loss, advanced clinical stage, CD4+ count, and decreased libido.

## 1. Background

Various studies, mainly from developed countries, have highlighted several endocrine disorders including hypogonadism as more prevalent in HIV infection in the precombined antiretroviral therapy (cART) era. These endocrine disorders were mainly associated with HIV-related opportunistic infections and advanced multiorgan failure [1–3]. Some of these disorders declined during the post-cART era but hypogonadism and diabetes remain frequent endocrinopathies in these patients [4]. Hypogonadism can have a significant clinical impact leading to sexual impairment, fatigue, depressed mood, anemia and weight loss, or muscle loss [1, 5, 6].

Hypogonadism can either be primary (hypergonadotropic) or secondary (hypogonadotropic). Luteinizing hormone and follicle-stimulating hormone measurements in addition to testosterone measurement will be required to differentiate them. Both primary and secondary hypogonadism are relatively common in people living with HIV/AIDS (PLHA), with secondary hypogonadism being more common [4, 7]. The exact cause of hypogonadism in HIV patients is not known, but decreased gonadotropin release from the pituitary is thought to be one of the mechanisms [8]. Hypogonadism can be intensified by various factors, and in addition, studies have proposed it to have multifactorial causes, including aging [5, 9], noninfectious comorbidities (obesity, diabetes, hypertension, cancers, and malnutrition among others) [5, 10–13], anemia [5, 11], common acute and chronic illnesses [14], weight loss [15], invasion of glands by HIV, or other pathogens such as hepatitis virus [16], cigarette smoking, using drugs, such as opiates, megestrol acetate, methadone [5, 17], and steroids [18], as well as progression to AIDS stages [19]. The use of herbal medicines could be one of the contributing factors to hypogonadism. Some herbal medicines are known to have antifertility properties by various mechanisms including inhibiting 5-alpha reductase, a factor that converts testosterone into dihydrotestosterone, reducing gonadotropins and testosterone secretion, and increasing the testosterone affinity for sex-specific proteins among others [20]. Studies have also reported that traditional herbal therapies and complementary alternative medicine are commonly used for the treatment of naïve HIV patients [21, 22] and therefore can be one of the cofactors for hypogonadism.

In Tanzania, despite the number of HIV patients remaining high and the reported occurrence of endocrinopathies in these patients, there is a paucity of information regarding the prevalence and risk factors of hypogonadism in these patients. Furthermore, screening for this condition is not routinely carried out in our setting. Therefore, the aim of this study was to determine the prevalence of hypogonadism and its associated risk factors among newly diagnosed ART naïve HIV-infected men in Mwanza, Tanzania.

## 2. Materials and Methods

### 2.1. Study Design, Setting, and Subjects

This was a cross-sectional study involving newly diagnosed male subjects before starting ART in Mwanza, Tanzania. Newly diagnosed HIV-positive (diagnosed as per WHO guidelines 2015) males aged 18 years and above attending at Voluntary Counseling and Testing (VCT) centres at Sekou-Toure Regional Referral Hospital (STRRH), Bugando Medical Centre (BMC), Nyamagana District Hospital (NDH), Magu District Hospital (MDH), and Ilemela District Hospital (IDH) were recruited and included in the study. Patients with previous history of gonadal dysfunction, taking drugs known to affect hormone levels (i.e., androgens, sex steroids, dehydroepiandrosterone, antiandrogens, antiandrogens, anabolic agents, GnRH agonists, and psycholeptic agents), and having chronic liver disease, chronic kidney injury, and chronic systemic illnesses such as tuberculosis and diabetes mellitus were excluded from this study.

The sample size for this study was calculated using Kish Leslie formula (1965). Using this formula, the required minimum sample size was found to be 296. A convenient sampling technique was used to enroll study participants, where subjects were recruited as they were coming at each institution until the sample size for the study was attained. At the completion of data collection, the distribution of participants was STRRH 105 (27.1%), BMC 110 (28.4), NDH 132 (34.0%, IDH 15 (3.8%), and MDH 26 (6.7%)

### 2.2. Data Collection and Laboratory Procedure

All individuals were assessed clinically by detailed history taking and general physical examination including anthropometric measurements that included waist circumference (WC) and body mass index (BMI). Sociodemographic data including age, employment status, marital status, and herbal medicine use status (whether they used any herbal medicine within the past six months or not) as well as symptoms of hypogonadism (whether experienced the symptoms in the past six months) were collected using a prestructured questionnaire. Height was measured in the upright standing position using a calibrated stadiometer. Body weight was measured with minimal clothing by using a standard calibrated weighing scale, and BMI was then calculated by the formula: weight in kilograms divided by height in meters squared. WC was measured at the approximate midpoint between the lower margin of the last palpable rib and the top of the iliac crest using flexible plastic tape and was calculated as an average of 3 measurements. Anthropometric parameters were measured by the same trained research assistant at each hospital.

Five milliliters (mls) of venous blood sample was collected from each of the study participants between 8.00 AM and 11.00 AM, and serum was harvested. The serum was used for the estimation of total testosterone (TT) hormone, follicle-stimulating hormone (FSH), luteinizing hormone (LH), and estradiol (E) levels. The serum samples were stored at −20°C for not more than 30 days until analyzed.

The hormonal tests were carried out using chemiluminescence immunoassay (CLIA) techniques. The CLIA kits were obtained from the Snibe Co., Ltd., Shenzhen, China. The fully auto chemiluminescence immunoassay analyzer model Maglumi 2000 (Snibe Diagnostic, China) was used to estimate serum hormones (TT, FSH, LH, and E) according to the principles of CLIA and protocols given by the kit manufacturers. The CD4+ count was assessed by flow cytometry (Roche Diagnostics).

Hypogonadism was defined as a serum TT level of <300 ng/dl or a serum TT level of ≥300 ng/dl with high FSH (>12 mlU/L) or LH (>12 mlU/L) level [4]. Eugonadism was defined as normal TT and normal FSH and LH levels. Compensatory hypogonadism was defined as normal TT but high FSH or LH levels. Primary hypogonadism was defined as low TT levels with high FSH and LH, while secondary hypogonadism was defined as low TT with low or normal FSH or LH [4, 23].

## 3. Data Analysis

Data were cleaned and checked for completeness and consistency and then corrected. The data were coded and entered into Microsoft Excel and then transported to STATA software, version 15 (Texas, USA) for analysis. Data were summarized using frequencies, percentages, or median with interquartile range (IQR). We used univariate followed by multivariate logistic regression models to determine factors associated with hypogonadism among newly diagnosed ART naïve HIV-infected males whereby factors with a *p* value less than 0.2 in the univariate were subjected to multivariate logistic regression analysis. Then, factors with a *p* value less than 0.05 were regarded as significantly associated with hypogonadism.

## 4. Results

### 4.1. Sociodemographic Characteristics of Study Participants

A total of 388 newly diagnosed ART naïve HIV-infected males were enrolled in this study of which 34% were from NDH and 28%, 27%, 6.7%, and 3.8% were from BMC, STRRH, MDH, and IDH, respectively. The median age of the participants was 40 (33–46) years (range 18–73 years), and the majority age group was 31–45 years (58.8%). The median BMI and WC of the study participants were 21.1 (19.4–23.5) kg/m^2^ and 80 (76–83) Cm, respectively. About three-quarters of participants had a BMI between 18.5 and 24.9 (normal), and more than fifty percent had a WC of less than 81 cm (lower). More than half, 63.7% (247/388), were married, about three quarters, 74.0% (287/388), were self-employed, and the majority, 67.0% (260/388), reported to have not used herbal medicine in the past six months (Table 1).

### 4.2. Clinical Characteristics and Symptoms of Hypogonadism among the Study Participants

Among 276 study participants who had CD4+ data, the median CD4+ count was 301.5 (169.0–410.5). The majority, 63.4% (245/388), of the study participants were grouped in stage one of the disease according to World Health Organization HIV staging. Regarding symptoms of hypogonadism, decreased libido was reported in 44.1% (171/388) of study participants, erectile dysfunction in 15.2% (59/388), and depression in 39.7% (154/388) while weight loss and fatigue were reported in 72.4% (281/388) and 76.6% (297/388) of study participants, respectively (Table 2).

### 4.3. Hormone Levels and Hypogonadism

**Figure 1:**
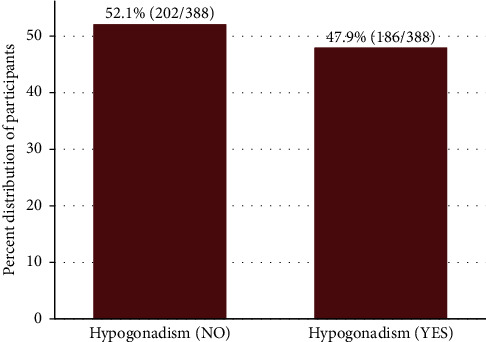
Prevalence of hypogonadism among study participants.

**Figure 2:**
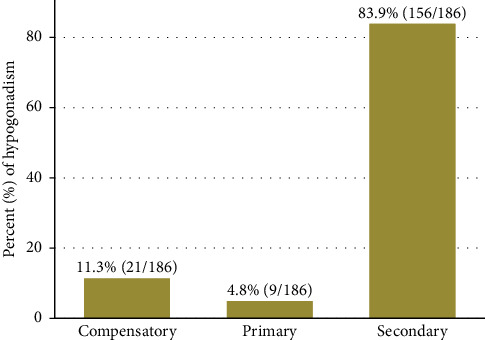
Categories of hypogonadism among participants with hypogonadism.

**Figure 3:**
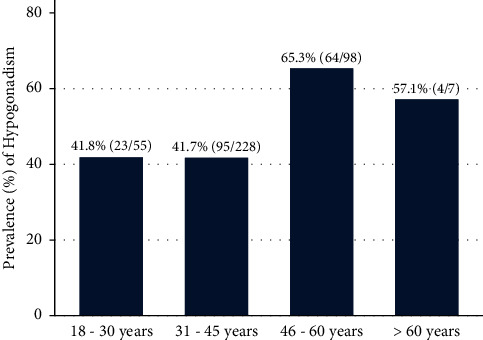
Distribution of hypogonadism prevalence according to the age group.

The median TT level was 390.5 (168.5– 560) ng/dl, the median LH level was 5.3 (4.0–7.4), and the median FSH level was 4.1 (2.5–6.5). Hypogonadism was seen in 186 (47.9%) (95% CI 43.0%–52.9%) of the study participants (Figure 1) whereby secondary hypogonadism was the most frequent type (83.9%, 156/186) while 4.8% (9/186) had primary and 11.3% (21/186) had compensatory hypogonadism (Figure 2). The prevalence of hypogonadism according to the age groups is shown in Figure 3. Hypogonadism was present even among participants below the age of 45 years with a prevalence of 42%.

### 4.4. Association of Hypogonadism with Sociodemographic and Clinical Characteristics

Following multivariate logistic regression analysis, the independent predictors of hypogonadism among newly diagnosed ART naïve HIV-infected males were age of above 46 years (OR 2.3; 95% CI 1.1–4.6; *p*=0.023), herbal medicine use (OR 2.4; 95% CI 1.5–3.9; *p* < 0.001), WHO clinical stage 3 (OR 2.7; 95% CI 1.4–5.2; *p*=0.003), and weight loss (OR 1.8; 95% CI 1.1–3.0; *p*=0.016). Univariate analysis showed a significant association between hypogonadism and CD4+ count (OR 2.0; 95% CI 1.1–3.6; *p*=0.022) and decreased libido (OR 1.6; 95% CI 1.1–2.4; *p*=0.024), but these factors were not subjected to multivariate logistic regression analysis because of collinearity with WHO clinical stage and age, respectively (Table 3).

## 5. Discussion

In this study, the prevalence of hypogonadism among ART naïve HIV-infected males was found to be 47.9% (95 CI 43.0%–52.9%). This rate of hypogonadism among ART naïve males is comparable with the rate in the previous studies conducted among ART naïve HIV-infected males by Dobs et al. (50%) [24] and Grispoon et al. (49%) [25] and lower than the finding by Tripathy et al. (89.7%) [26] and higher than the finding by Raffi et al. (29%) [27] but generally higher than the finding of other studies among HIV males on ART reporting prevalence ranging from 16 to 34 [1, 15]. A recent meta-analysis study by Santi et al. reported a prevalence of 33.0% when total testosterone alone was considered, but most studies employed were carried out among HIV patients on ART and used a different cutoff of testosterone including a lower value than the current cutoff of 300 ng/dl [28]. The heterogeneity of hypogonadism prevalence reported in the ART era is due to differences among studies in terms of serum TT assays, use of cFT or TT, use of different cutoffs, and mean age of patients [28, 29]. During the pre-ART era, studies have shown a high prevalence of hypogonadism, approximately 50% with AIDS, which is associated with increased severity of the disease [27]. The use of cART leading to a lower prevalence of hypogonadism in the post-cART era is expected to be related to the reduction in the number of patients with advanced HIV/AIDS. However, hypogonadism remains a significant problem even among patients in the early stages of HIV infection, particularly when the prevalence is still higher than the average rate for the general population [1]. Studies showed the prevalence of hypogonadism in the general population ranged from 2.1% to 12.8% in middle aged to older men [30]. The present study showed that the prevalence of hypogonadism in ART naïve HIV-infected males was higher than that of the general population. In this study, the prevalence of hypogonadism was 42% among HIV-infected males below 45 years. In a study by Rochira et al. among HIV-infected men on HAART in Italy, the highest rate of hypogonadism was seen in men aged 40–49 (15.3%, 123/800) and 50–59 (23%, 58/245) years. Notably, 10.6% of patients in the age group 30–39 years also had hypogonadism [4].

Among men with hypogonadism, secondary hypogonadism was the predominant form. This finding is similar to observations by several authors. Rochira et al. [4] reported secondary hypogonadism in 86 percent of hypogonadal patients with testosterone <300 ng/dl. Similarly, in the study by Pongener et al. [31] and Dutta et al. [32], hypogonadotropic hypogonadism was found in 86% and 81% of hypogonadal patients, respectively. This observation could be due to invasion of the hypothalamic pituitary axis by the virus leading to impairment of its function.

Remarkably, the present study, different from most of the previous studies, reported the presence of hypogonadism and/or reduced testosterone in ART naïve patients (not exposed to ART), therefore indicating that the virus itself or the comorbid condition is able to influence the hypothalamic-pituitary-gonadal axis. Previous studies have shown that poor health status is associated with worse gonadal function in HIV patients [10, 33]. Secondary hypogonadism might be due to inhibition of gonadotropin secretion through a mechanism such as leptin resistance or adipokine release [34, 35] as a result of the effect of the virus itself or comorbid conditions.

Our study found a significant association between hypogonadism and older age. This finding corresponds to the previous report which showed increasing prevalence with age [36, 37]; however, in HIV, hypogonadism also tends to occur at an earlier age affecting young-aged and middle-aged men. About three-quarter of our study participants (72.9%) were between the ages of 18 and 45 years, and 42% of them had hypogonadism.

In our study, we did not observe an association between BMI and hypogonadism. Similar to our findings, Klein et al. also did not find a significant association between low androgen levels with BMI [9]. Furthermore, some other studies did not find any association with weight [15, 25]. However, different from our observation, a study by Crum-Cianflone et al. demonstrated a link between increasing BMI and hypogonadism [7]. In studies done by Meena et al. [38] and Jain et al. [19], the incidence of low testosterone was directly associated with BMI. The lack of association between BMI and hypogonadism in our study could be due to the smaller number of patients with higher BMI. Most of the study participants in the current study had normal BMI, and only a few were obese or overweight. The higher rate of participants with normal BMI and above recorded in our study could be due to regional variations and the fact that patients with debilitating chronic/systemic diseases were excluded from the study.

A statistically significant association was also found between the WHO clinical stage (that determined the clinical progression of the disease) and hypogonadism. HIV infection causes impaired immunity with an associated decrease in CD4+ count leading to comorbid conditions, hence poor health status [10]. This observation may be explained by the link between poor health status and impaired gonadal function [28]. Furthermore, we found that male hypogonadism was associated with weight loss. This is in support of the finding by Rietschel et al. [15] which showed hypogonadism to be more common among patients with AIDS-associated wasting.

Limitations of our study include the use of TT to diagnose hypogonadism which can underestimate the prevalence of hypogonadism due to the possible rise in serum SHBG in HIV patients. Measurement of SHBG has been highly recommended, in addition to serum LH and TT in these patients [39, 40]. Another limitation is that testosterone levels were determined using an immune-assay technique, whereas mass spectroscopy is often considered “gold standard” but is not commonly used because it is expensive and not widely available. However, the immuno-chemiluminescence assay used in the determination of gonadal hormone values is internationally certified and widely used in clinical practice to diagnose and guide treatment in patients with gonadal dysfunction.

## 6. Conclusion

Nearly half of ART naïve HIV-infected men were found to have hypogonadism with 42% of patients below 45 years of age. Four in every five ART naïve HIV-infected men with hypogonadism had a secondary type suggesting the hypothalamic-pituitary axis could be the main element involved in the development of hypogonadism in HIV patients. There was a significant association of hypogonadism with older age, herbal medicine use, weight loss, and advanced clinical stage.

## Figures and Tables

**Table 1:** Sociodemographic characteristics of 388 study participants.

Variable	Frequency (*n*)/median	Percent (%)/IQR
Median age (years)	40	(33–46)
Age groups (years)		
18–30	55	14.2
31–45	228	58.8
46–60	98	25.3
>60	7	1.8
Median BMI (kg/m^2^)	21.1	(19.4–23.5)
BMI category (kg/m^2^)		
<18.5	56	14.4
18.5–24.9	277	71.4
25–29.9	51	13.1
≥30	4	1.0
Median waist circumference (cm)	80	(76–83)
Waist circumference category (cm)		
<81	214	55.2
81–93	162	41.8
94–115	12	3.1
Marital status		
Married	247	63.7
Single	86	22.2
Divorced/separated	55	14.2
Herbal medicine use		
Yes	128	33.0
No	260	67.0
Employment status		
Non-self-employed	75	19.3
Self-employed	287	74.0
Unemployed	26	6.70

**Table 2:** Reported symptoms of hypogonadism and clinical characteristics of the 388 study participants.

Symptom/clinical characteristics	Frequency (*n*)/median	Percent (%)/IQR
Median CD4+ count^*∗*^	301.5	(169.0–410.5)
CD4+ count category^*∗*^		
<200	80	29.0
200–350	85	30.8
>350	111	40.2
Clinical stage		
Stage 1	246	63.4
Stage 2	58	15.0
Stage 3	62	16.0
Stage 4	22	5.7
Decrease libido		
Yes	171	44.1
No	217	55.9
Erectile dysfunction		
Yes	59	15.2
No	329	84.8
Depression		
Yes	154	39.7
No	234	60.3
Weight gain		
Yes	6	1.5
No	382	98.5
Weight loss		
Yes	281	72.4
No	107	27.6
Fatigue		
Yes	297	76.5
No	91	23.5
Breast enlargement		
Yes	3	0.8
No	385	99.2

^*∗*^Number of participants *N* = 276.

**Table 3:** Factors associated with hypogonadism among newly diagnosed ART naïve HIV-infected adult males.

Factor/variable	Hypogonadism		Univariate		Multivariate	
	Yes *n*(%)	No *n*(%)	OR (95% CI)	*p* value	OR (95% CI)	*p* value
Age (years)						
18–30	23 (41.8)	32 (58.2)	1.0		1.0	
31–45	95 (41.7)	133 (58.3)	1.0 (0.5–1.8)	0.984	0.8 (0.5–1.6)	0.598
>46	68 (64.8)	37 (35.2)	2.6 (1.3 5.0)	0.006	2.3 (1.1–4.6)	0.023
BMI						
18.5–24.9	131 (47.3)	146 (52.7)	1.0		1.0	
<18.5	32 (57.1)	24 (42.9)	1.5 (0.8–2.7)	0.180	1.1 (0.6–2.0)	0.819
25–29.9	21 (41.2)	30 (58 0.8)	0.8 (0.4–1.4)	0.422	0.8 (0.4–1.6)	0.515
≥30 9	2 (50.0)	2 (50.0)	1.1 (0.2–8.0)	0.914	1.7 (0.2–13.6)	0.607
Waist circumference						
81–93	81 (50.0)	81 (50.0)	1.0			
<81	96 (44.9)	118 (55.1)	0.8 (0.5–1.2)	0.323	—	—
94–115	9 (75.0)	3 (25.0)	3.0 (0.8–11.5)	0.109	—	—
Marital status						
Married	121 (49.0)	126 (51.0)	1.0			
Single	39 (45.4)	47 (54.6)	0.9 (0.5–1.4)	0.561	—	—
Divorced	26 (47.3)	29 (52.7)	0.9 (0.5–1.7)	0.818	—	—
Herbal medicine use						
No	104 (40.0)	156 (60.0)	1.0		1.0	
Yes	82 (64.1)	46 (35.9)	2.7 (1.7–4.1)	<0.001	2.4 (1.5–3.9)	<0.001
CD4+ count						
>350	41 (36.9)	70 (63.1)	1.0			
<200	43 (53.7)	37 (46.3)	2.0 (1.1–3.6)	0.022^*∗*^	—	—
200–350	42 (49.4)	43 (50.6)	1.7 (0.9–3.0)	0.081	—	—
Clinical stage						
Stage 1	97 (39.4)	149 (60.6)	1.0		1.0	
Stage 2	30 (51.7)	28 (48.3)	1.6 (0.9–2.9)	0.089	1.7 (0.9–3.1)	0.081
Stage 3	44 (71.0)	18 (29.0)	3.8 (2.1–6.9)	<0.001	2.7 (1.4–5.2)	0.003
Stage 4	15 (68.2)	7 (31.8)	3.3 (1.3–8.4)	0.012	2.5 (0.9–6.6)	0.072
Decrease libido						
No	93 (54.4)	124 (45.6)	1.0			
Yes	93 (42.9)	78 (57.1)	1.6 (1.1–2.4)	0.024^*∗*^	—	—
Erectile dysfunction						
No	155 (47.1)	174 (52.9)	1.0			
Yes	31 (52.5)	28 (47.5)	1.2 (0.7–2.2)	0.443	—	—
Depression						
No	111 (47.4)	123 (52.6)	1.0			
Yes	75 (48.7)	79 (51.3)	1.1 (0.7–1.6)	0.807		
Weight loss						
No	39 (36.4)	68 (63.6)	1.0		1.0	
Yes	147 (52.3)	134 (47.7)	1.9 (1.2–3.0)	0.006	1.8 (1.1–3.0)	0.016
Fatigue						
No	41 (45.1)	50 (54.9)	1.0			
Yes	152 (51.2)	145 (48.8)	1.2 (0.7–1.9)	0.529	—	—
Breast enlargement						
No	184 (47.8)	201 (52.2)	1.0			
Yes	2 (66.7)	1 (33.3)	2.2 (0.2–24.3)	0.525	—	—

^*∗*^These factors were not subjected to multivariate logistic regression analysis because of collinearity with WHO clinical stage and age, respectively (*N* = 388).

## Data Availability

The datasets used and/or analyzed during the current study are available from the corresponding author on reasonable request.
